# Microbial methane formation in deep aquifers of a coal-bearing sedimentary basin, Germany

**DOI:** 10.3389/fmicb.2015.00200

**Published:** 2015-03-20

**Authors:** Friederike Gründger, Núria Jiménez, Thomas Thielemann, Nontje Straaten, Tillmann Lüders, Hans-Hermann Richnow, Martin Krüger

**Affiliations:** ^1^Resource Geochemistry, Geomicrobiology, Federal Institute for Geosciences and Natural Resources, HannoverGermany; ^2^Federal Institute for Geosciences and Natural Resources, HannoverGermany; ^3^Institute of Groundwater Ecology, Helmholtz Center for Environmental Health, NeuherbergGermany; ^4^Department of Isotope Biogeochemistry, Helmholtz Centre for Environmental Research, LeipzigGermany

**Keywords:** methanogenesis, Cenozoic sediments, fluvial deposits, stable isotope fractionation, methanogenic hydrocarbon degradation, methanogenic archaea, coal

## Abstract

Coal-bearing sediments are major reservoirs of organic matter potentially available for methanogenic subsurface microbial communities. In this study the specific microbial community inside lignite-bearing sedimentary basin in Germany and its contribution to methanogenic hydrocarbon degradation processes was investigated. The stable isotope signature of methane measured in groundwater and coal-rich sediment samples indicated methanogenic activity. Analysis of 16S rRNA gene sequences showed the presence of methanogenic *Archaea*, predominantly belonging to the orders *Methanosarcinales* and *Methanomicrobiales*, capable of acetoclastic or hydrogenotrophic methanogenesis. Furthermore, we identified fermenting, sulfate-, nitrate-, and metal-reducing, or acetogenic *Bacteria* clustering within the phyla *Proteobacteria*, complemented by members of the classes *Actinobacteria*, and *Clostridia*. The indigenous microbial communities found in the groundwater as well as in the coal-rich sediments are able to degrade coal-derived organic components and to produce methane as the final product. Lignite-bearing sediments may be an important nutrient and energy source influencing larger compartments via groundwater transport.

## Introduction

The occurrence of methane in coal-bearing sedimentary basins has traditionally been linked to high maturity coals with thermogenic gas potential (Strąpoć et al., 2011). However, recent geochemical studies have frequently detected natural occurrence of biogenic methane, identified by its carbon and hydrogen isotopic signatures (Whiticar et al., 1986), in groundwater and pore water from lower maturity coal systems, like those in the Powder River Basin (Strąpoć et al., 2011 and the references therein). Biogenic methane can be generated from coal material under anoxic conditions (Krüger et al., 2008; Beckmann et al., 2011a; Guo et al., 2012). Coal is extremely rich in complex organic matter and therefore a very attractive carbon source for microbial biodegradation (Fakoussa and Hofrichter, 1999) and, eventually, for methanogenesis. Lignite coal is soft brown sediment with high water content and a relatively low heating value. These characteristics place it somewhere between peat and sub-bituminous coal. Because of the high organic matter concentrations, coal-derived sediments are potential important microbial sources of energy (Fry et al., 2009). The biodegradation of coal components is primarily performed by bacterial fermentation of polymers and monomers to fatty acids, organic acids, alcohols and/or hydrogen and carbon dioxide. These products can then be used by methanogens. Thus, the successful conversion of coal to methane depends on the syntrophic interaction of both microbial groups: fermenting bacteria and methanogenic *Archaea* (For a review see Strąpoć et al., 2011).

Based on laboratory methanogenesis stimulation studies and the analysis of archaeal communities (where members of *Methanosarcinales*, belonging to *Methanosarcina* and *Methanosaeta*, were abundant), Krüger et al. (2008) reported a predominance of acetoclastic methanogenesis in hard coal and timber in abandoned mines. These results were later confirmed by Stable Isotope Probing (Beckmann et al., 2011b). In a subsequent study, Beckmann et al. (2011a) described the bacterial community present in those mines, dominated by potential fermenters and acetogens.

Other studies conducted in the Gippsland Basin (Midgley et al., 2010), the Illinois Basin (Strąpoć et al., 2008), Northern Japan (Shimizu et al., 2007) and Western Canada (Penner et al., 2010), reported the presence not only of *Methanosarcina* and *Methanosaeta*, but also of several other methanogenic archaeal genera including *Methanolobus*, *Methanobacterium*, *Methanocorpusculum*, *Methanococcus*, *Methanoculleus*, and *Methanoregula* in formation water samples and sediment cores. Dominant bacterial species included the phyla *Firmicutes*, *Spirochaetes*, *Bacteroidetes* and members of all subgroups of *Proteobacteria* (Strąpoć et al., 2011). Detmers et al. (2001) showed the presence of an anaerobic food chain in groundwater from a pristine aquifer with lignite layers, consisting of active fermenting *Betaproteobacteria* and sulfate-reducing bacteria (*Desulfotomaculum spp.*) at the interface between aquifer and lignite seam.

Nevertheless, to date, little is known about the biodegradation processes of coal leading to methane production in ligniteous coal-rich sediments and coal-associated aquifers, the diversity of the microbial communities involved, and the influence of geochemical parameters. Because of this, the aim of this work was (1) to study the importance of coal-derived organic substrates for methanogenic microbial communities present in sediment and formation water samples, (2) to investigate microbial syntrophic interactions and degradation pathways and (3) the microorganisms involved in the biodegradation. This should contribute to a better understanding of metabolic processes in coal-associated habitats leading to biogenic gas generation. For this purpose, we sampled groundwater and coal-rich sediments from a coal-associated sedimentary basin, where isotopic signatures of methane (δ^13^C-CH_4_ and δD-CH_4_) indicated the occurrence of biogenic methane. Geochemical investigations were combined with microbiological and molecular biological approaches leading to the identification and characterization of the bacterial and archaeal community composition in coal-rich sediment and groundwater samples as well as methanogenic enrichment cultures with hydrocarbons as sole carbon source. Integrating these methods we were able to show a close interaction between organic substrates and microbial populations in coal-rich sediments with the groundwater aquifer system.

## Materials and Methods

### Sampling and Sample Preparation

Groundwater samples and samples of coal-bearing sediments were collected in February 2009. Groundwater samples were taken from 10 different continuously running wells from the groundwater management system in the proximity of an open-cast lignite coal mine. They were collected in sterile bottles previously flushed with N_2_. Bottles were completely filled with water and caped with butyl rubber stoppers to prevent further O_2_ exposure. All samples were transported and stored cool (4°C) and processed 1 day after sampling. The main geochemical properties for each one are given in **Table 1**. All groundwater samples showed pH-values of ∼7, the salinity ranged between 5 and 9%. The locally measured water temperature was ∼29°C, only in well site 3 a water temperature of 15°C was detected, the air temperature ∼0°C. Four groundwater samples (from wells 2, 4, 5, 10) smelled sulfurous, the other were odorless. For the investigation of the water chemistry, the fluids were immediately filtered upon arrival (0.45–0.22 μm depending on the analysis) and stored at 4°C or frozen (-20°C) until further analysis.

**Table 1:** Methane content and geochemical properties of groundwater samples collected from wells located in coal-rich sediments.

Water	CH_4_-content	pH-	EC	Na^+^	Cl^-^	SO_4_^2-^	HCO_3_^-^	Fe^2+^	Mn^2+^	NO_3_^-^	NH_4_^+^	PO_4_^3-^	TIC	NPOC
site	[μM]	value	[μS/cm]	[mg/l]	[mg/l]	[mg/l]	[mg/l]	[mg/l]	[mg/l]	[mg/l]	[mg/l]	[mg/l]	[mg/l]	[mg/l]
Well 1	10.2	6.8	471	63.9	31.4	1.73	242	0.459	0.039	0.27	0.64	0.45	64	3.1
Well 2	24.2	6.9	691	111	34.8	1.04	398	0.282	0.056	0.09	0.66	0.56	100	4.9
Well 3	22.1	6.8	558	55.2	16.9	14.2	325	1.95	0.191	0.02	0.39	0.90	89	2.3
Well 4	37.5	7.3	616	90.7	27.3	0.54	359	0.302	0.034	0.03	0.66	0.49	90	4.1
Well 5	99.4	7.1	848	150	28.7	1.12	523	0.951	0.052	0.01	0.79	0.72	133	7.1
Well 6	51.0	7.2	699	131	27.8	0.84	417	0.364	0.053	0.02	0.72	0.69	108	6.1
Well 7	13.4	6.9	423	53.3	19.7	2.16	237	0.234	0.071	0.02	0.5	0.42	67	2.9
Well 8	71.3	7.1	742	169	21.2	0.95	456	0.299	0.011	0.03	0.6	1.29	117	11.4
Well 9	16.5	6.9	485	61.0	22.0	3.24	273	0.272	0.056	0.02	0.54	0.36	73	2.9
Well 10	24.9	7.3	551	81.5	29.8	0.87	304	0.124	0.056	0.04	0.57	0.52	82	4.6

EC, electrical conductivity; TIC, total inorganic carbon; NPOC, non purgeable organic carbon.

In addition, three coal-rich sediment samples were collected from freshly mined heaps of brown coal (sample 1), air-dried coal from the bottom of the mine (sample 2) and coal slurry from a wet spot (sample 3). Collected groundwater and coal-rich sediment samples were transferred into sterile glass bottles and immediately flushed with N_2_. Directly after collection of groundwater samples, pH, temperature, conductivity and salinity were determined. All samples were transported and stored at 4°C for further analyses.

### Cultivation Methods

Anaerobic incubations were set up in an anaerobic chamber. A first set of incubations was established to determine methanogenic and sulfate-reducing rates. Three g of sediment (sediment microcosms) or 10 mL of groundwater (groundwater microcosms) were transferred into autoclaved 19-mL Hungate vials containing 5 mL of freshwater medium (Widdel and Bak, 1992) with 2 mM (in the case of the methanogenic microcosms, to stimulate the first biodegradation steps, according to Zengler et al., 1999) or 10 mM sulfate (for the sulfate-reducing ones). The glass vials were sealed with sterile butyl rubber stoppers and aluminum crimp caps. All tubes were flushed with N_2_ to remove traces of H_2_ from the anaerobic chamber.

To investigate methane production rates related to different methanogenic degradation pathways, cultures (three of each) were amended with either acetate (10 mM), methanol (0,5 mM) or a H_2_/CO_2_ mix (80/20%). Controls without any added carbon source were incubated in parallel. Cultures with 2-bromoethanesulfonate (BES; 10 mM), a specific inhibitor for methanogenic microorganisms, were included as well to account for possible non-microbial methane emissions from the water or sediment samples. Sulfate-reducing microcosms, containing either lactate or acetate (10 mM of each) or without any additional substrate (three of each), were set up. In this case, cultures with sodium azide (NaN_3_, 50 mM), a strong microbial toxin, were used as controls to show feasible degassing from non-microbial origin.

All microcosms were incubated at 30°C in the dark and monthly sampled to assess methane and CO_2_ formation in the headspace. Methane and CO_2_ production rates were calculated by linear regression of each gas increased with incubation time and expressed in μmol day^-1^ mL^-1^ groundwater or μmol day^-1^ gDW^-1^ (dry weight) of sediment (Krüger et al., 2001). Sulfate-reduction rates were followed via the production of copper sulfide from dissolved sulfide (HS^-^), according to Cord-Ruwisch (1985).

After 94 days of incubation, growing methanogenic cultures (5 mL) were subsequently re-inoculated into fresh medium and amended with ^13^C-labeled substrates ([^13^C_16_] *n*-hexadecane, [^13^C_7_] toluene, [^13^C_2_] ethylbenzene, or 2-[^13^C]-methylnaphthalene), to investigate the transformation of selected hydrocarbons into methane and CO_2_. ^13^C-labeled or unlabeled single substrates, *n*-hexadecane, ethylbenzene (both 0.1% v/v), toluene or 2-methylnaphthalene (0.5 mg of each), were added into the anaerobic enrichment cultures, containing 25 mL fresh sterile medium and 5 mL transferred pre-culture from groundwater or coal-rich sediment samples (as described previously) in 56-mL serum bottles. All labeled or unlabeled hydrocarbons (ethylbenzene, toluene, and methylnaphthalene) were from Campro Scientific GmbH (Germany), except the U-^13^C-*n*-hexadecane, which was synthesized as described by Feisthauer et al. (2010).

### Analytical Methods

The elemental composition of 10 different groundwater samples was analyzed using an inductively coupled-plasma mass-spectrometry instrument (ICP-MS ELAN 5000, Perkin Elmer Sciex, USA; Dekov et al., 2007). Concentrations of potassium, sodium, chloride, magnesium, calcium, sulfate, bicarbonate, ferrous iron, manganese, aluminum, arsenic, borate, barium, cadmium, chromium, lithium, nickel, lead, silica, and strontium were measured. Anions (nitrite, nitrate, and phosphate) were determined by ion chromatography with a DX-500 ion chromatograph system (Dionex, Germany) and ammonium was detected by flow injection analysis according to DIN EN ISO 11732. Total inorganic carbon (TIC) and dissolved organic carbon (DOC; 0.45 μm filtered) were measured using catalytic high temperature combustion with a Shimadzu TOC-VCPN carbon analyzer (Shimadzu, Japan).

Methane and CO_2_ concentrations from groundwater samples and from microcosms headspace were analyzed using a methanizer-equipped gas chromatograph with flame ionization detector (GC-FID) fitted with a 6^′^ Hayesep D column (SRI 8610C, SRI Instruments, USA) running isothermally at 60°C, after reduction of CO_2_ to methane. Carbon and hydrogen isotopic signatures from methane and carbon dioxide emanated from the coal-rich sediment samples in the bottles were determined using a gas-chromatography-combustion-isotope ratio mass spectrometry system (GC-C-IRM-MS), equipped with a CP-pora BOND Q column coupled to a combustion or high temperature pyrolysis interface (GC-combustion III or GC/C-III/TC; Thermo Finnigan, Bremen, Germany) and a MAT 252 IRMS for the carbon analysis or a MAT 253 IRMS for the hydrogen analysis (both from Thermo Finnigan, Bremen, Germany; Feisthauer et al., 2010; Herrmann et al., 2010). The carbon and hydrogen isotopic compositions (R) are reported as delta notation (δ^13^C and δD) in parts per 1000 (‰) relative to the Vienna Pee Dee Belemnite (VPDB) and Vienna Standard Mean Ocean Water (VSMOW), respectively. The error associated with the system (accuracy and reproducibility) was around 0.5‰ for carbon and 4‰ for hydrogen.

### Molecular Biological Methods

#### Quantification of Microorganisms in Environmental Samples and Cultures

Total cell numbers were counted after staining with SYBR Green II under the fluorescence microscope as described by Weinbauer et al. (1998).

Genomic DNA from the coal-rich sediments and from the microcosms amended with hydrocarbons was extracted using protocols from Lueders et al. (2004). Groundwater samples were aseptically filtered with membrane filters (0.22 μm; Whatman, General Electric Company, Munich, Germany) and DNA was extracted from the filters according to Lueders et al. (2004).

16S rRNA gene copy numbers of *Archaea* and *Bacteria* were determined as described previously (Takai and Horikoshi, 2000; Nadkarni et al., 2002) using the Q-PCR instrument ABI Prism 7000 (Applied Biosystems, Life Technologies Corporation, USA). In addition, Crenarchaeota were quantified according to Ochsenreiter et al. (2003). Concentrations of methyl-coenzyme M reductase subunit alpha gene (*mcrA*; using mlas and mcrA-rev primers) and *dsrA* gene coding for the alpha subunit of the dissimilatory (bi)sulfite reductase of sulfate-reducing prokaryotes were determined according to Steinberg and Regan (2008, 2009) and Schippers and Neretin (2006). All Q-PCR reactions were measured in three parallels and three dilutions, to account for possible inhibitor effects in the DNA extracts. To perform Q-PCR quantification, a StepOne detection system (StepOne version 2.0, Applied Biosystems, USA) coupled with the StepOne v2.1 software was used.

#### Terminal Restriction Fragment Length Polymorphism

For terminal restriction fragment length polymorphism (T-RFLP) analysis, extracted DNA was used as template for PCR amplification of phosphoramidite fluorochrome 5-carboxyfluorescein (FAM)-labeled amplicons. Amplifications were generated with the use of the primer sets Ar109f and 912rt-FAM, or Ba27f-FAM and 907r. To account for possible inhibitor effects in environmental DNA extracts, a dilution series of each fresh extract was used. T-RFLP analysis of PCR products was done using the restriction endonucleases TaqI (archaeal assay) and MspI (bacterial assay), respectively. The procedure was described by Pilloni et al. (2011). Capillary electrophoresis and data collection were operated on an ABI 3730 Genetic Analyzer (Applied Biosystems, USA). The electropherograms were processed with sequence analysis software PeakScanner 1.0 and GeneMapper 4.0 (Applied Biosystems, USA). T-RFLP histograms were performed with the use of the T-REX online software using the default settings (Culman et al., 2009). Terminal restriction fragments were compared to theoretical predictions from 16S rRNA gene sequences for a preliminary identification of specific groups of bacteria. The particular T-RF length represents the most abundant microorganisms within the bacterial community.

#### Clone Libraries

Clone libraries were created using DNA extract from the original coal-rich sediment samples and the derived microcosms amended with hydrocarbons. 16S rRNA gene fragments were amplified by PCR using the domain specific primer pairs 21f (5^′^-TTC CGG TTG ATC CYG CCG GA) and 958r (5^′^-YCC GGC GTT GAM TCC AAT T) for Archaea (DeLong, 1992), and GM3f (5^′^-AGA GTT TGA TCM TGG C) and GM4r (5^′^-TAC CTT GTT ACG ACT T) for Bacteria (Lane, 1991). Cloning and sequencing of the archaeal and bacterial 16S rRNA amplicons was performed by Microsynth AG^1^ (Switzerland). Sequences were assembled using the Geneious ProTM 5.3 software^2^. Prior to phylogenetic analysis, vector sequences flanking the 16S rRNA gene inserts were removed. Chimeric sequences were detected using the DECIPHER’s Find Chimeras online software (Wright et al., 2012) from the University of Wisconsin Madison^3^ and were excluded from further analysis. Sequences were compared to GenBank BLASTn algorithm from the National Center for Biotechnology Information (Altschul et al., 1990)^4^ and the Ribosomal Database Project Classifier (Wang et al., 2007; RDP^5^) to select closely related species. Sequences were aligned with their nearest neighbors in the SSU dataset using SINA Alignment Service^6^ (Pruesse et al., 2012).

#### Amplicon Pyrosequencing

Amplicon pyrosequencing of bacterial 16S rRNA genes was performed on a 454 GS FLX Titanium system (Roche, Penzberg, Germany) as reported by Pilloni et al. (2012). Briefly, bar-coded amplicons for multiplexing were prepared using the primers Ba27f and Ba519r (for an easier linking of observed TRFs to restriction sites predicted for assembled pyrotag contigs) and extended with the respective adapters, key sequence and multiplex identifiers (MIDs) as recommended by Roche. Pyrotag PCR was performed in a Mastercycler ep gradient (Eppendorf, Hamburg, Germany) as described in Pilloni et al. (2012), and amplicons were subsequently purified.

Quality filtering of the pyrosequencing reads was performed using the automatic amplicon pipeline of the GS Run Processor (Roche) with a modification of the valley filter (vfScanAll- Flows false instead of TiOnly) to extract sequences. Reads were further trimmed using the TRIM function of GREENGENES (DeSantis et al., 2006) with default settings, and those shorter than 250 bp or with incorrect sequencing primers were excluded from further analysis. Read affiliation was done for combined forward and reverse reads for each library using the RDP classifier (Wang et al., 2007) with confidence threshold set to 80% (default).

Contigs for T-RF prediction of dominating amplicons were assembled with using SEQMAN II software (DNAStar, Madison, WI, USA), using forward- and reverse-reads, as described elsewhere (Pilloni et al., 2012). Thresholds of read assembly into one contig were set to at least 98% sequence similarity for a minimum overlap of 50 bp. Contigs within one library with less than 20 reads and not at least one forward and one reverse read were excluded from further analysis.

Cloning sequences and contigs originated from pyrosequencing were grouped into operational taxonomic units (OTUs) based on a sequence similarity cutoff of 97% (Yu et al., 2006) using Mothur software^7^ (Schloss et al., 2009). One clone sequence from each OTU having two or more representatives was submitted to NCBI GenBank database (accession numbers KJ424433 to KJ425107).

## Results

### Occurrence of Biogenic Methane in Groundwater and Sediments

The high TIC content in all groundwater samples, demonstrated the presence of active CO_2_-releasing microbial metabolization processes (**Table 1**). Dissolved CH_4_ concentrations ranged from 10 μM in well 1–100 μM in well 5 (**Table 1**).

**FIGURE 1:**
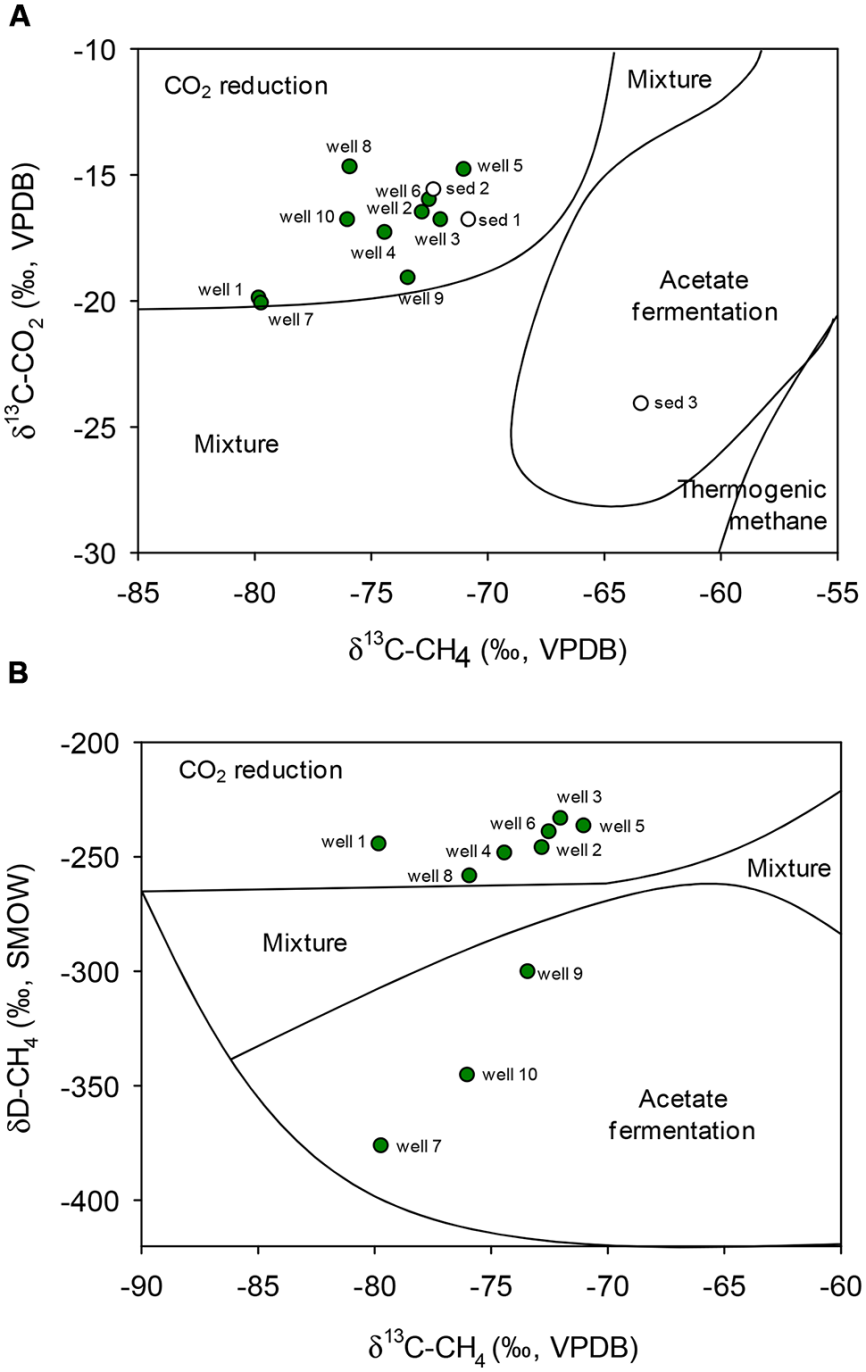
**Genetic diagrams for gasses of different samples: carbon isotopic composition of natural methane and CO_2_ in groundwater samples from deep aquifers of coal-rich sedimentary basin and sediments **(A)**.** Hydrogen vs. carbon isotopic composition of methane (δ^13^C_CH4_ and δD_CH4_) of natural methane in groundwater samples **(B)**. Zoning after Schoell (1980, 1983) and Whiticar et al. (1986). VPDB, Vienna PeeDee Belemnite; SMOW, Standard Mean Ocean Water.

Dissolved methane and CO_2_ exhibited light carbon isotopic signatures, with δ^13^C values ranging from -71 to -80‰ and -14 to -20‰ relative to VPDB, respectively (**Table 1**). These values are on the lower range of previously reported δ^13^C signatures for coalbed methane (-80 to -17‰) and CO_2_ (-27 to+19‰) as compiled by Rice (1993), and are compatible with a biogenic origin of the methane (**Figure 1A**). The isotopic composition of methane hydrogen δD-CH_4_ was highly variable but relatively light, ranging from -234 (well 3) to -376‰ (well 7), consistent with either CO_2_ reduction or bacterial methyl-type fermentation (**Figure 1B**; Schoell, 1980, 1983; Whiticar et al., 1986; Whiticar, 1999).

Similarly, C isotopic composition of natural gas collected from coall-rich sediments was light, with δ^13^C-CH_4_-values of -70.8‰ (sediment 1), -72.3‰ (sediment 2) and -63.4‰ (sediment 3), and δ^13^C-CO_2_-values of -16.8, -15.6, and - 24.1‰, respectively. These signatures were consistent with a biogenic origin as well, either by CO_2_ reduction (sediments 1 and 2) or acetate fermentation (sediment 3; **Figure 1A**).

### *In vitro* Methanogenic and Sulfate Reduction Rates

**FIGURE 2:**
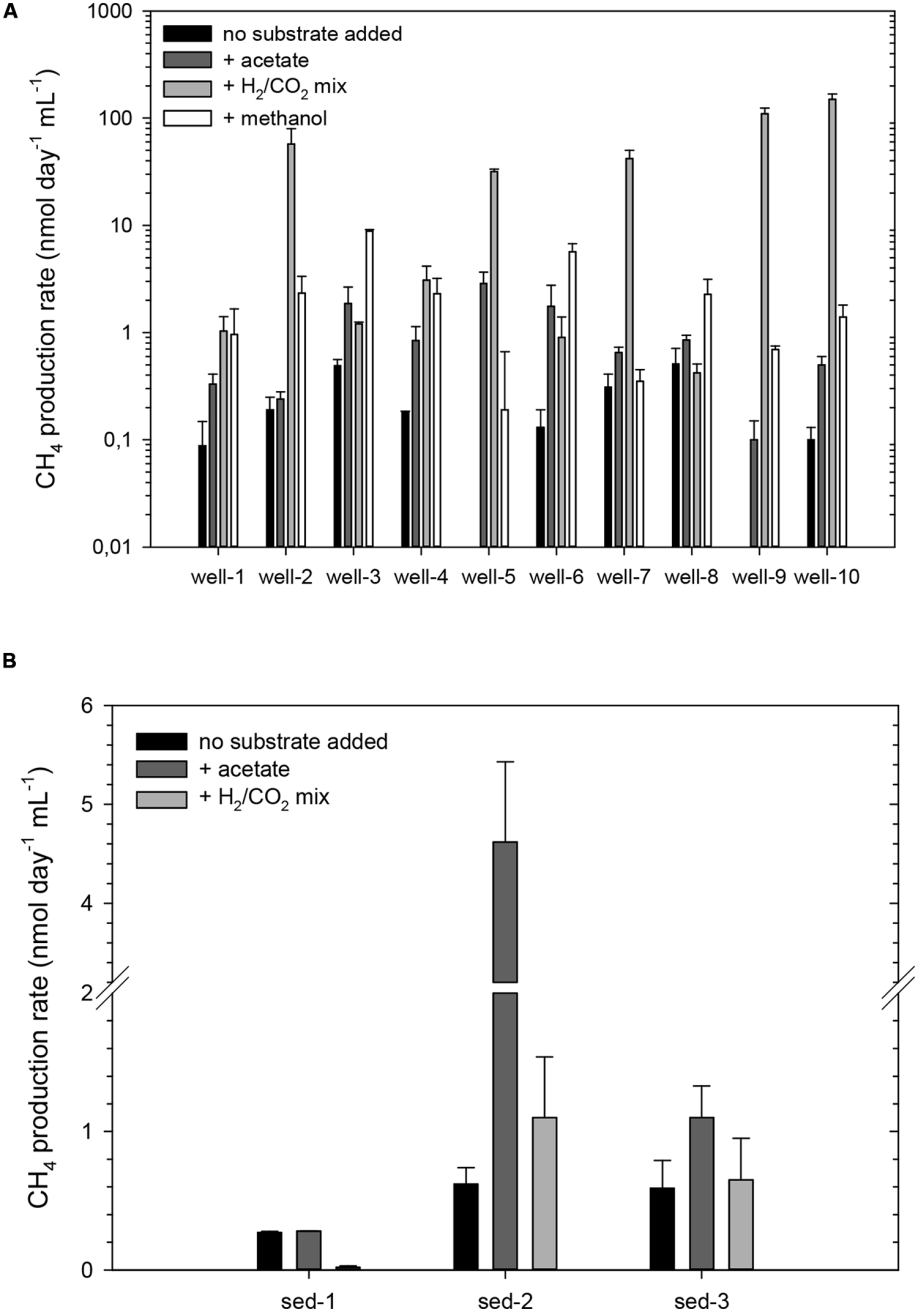
**Methane production rates from cultured groundwater samples from wells 1 to 10 amended without any additional substrate, or with either acetate, a mixture of H_2_ and CO_2_ or methanol **(A)**, and from sediments 1–3 without any substrate added or amended with either acetate, or a mixture of H_2_ and CO_2_**(B)**.** Notice the different scale of the Y axis. The error bars represent the SD of three replicates. All cultures were incubated at 30°C for 73 days.

Groundwater and coal-rich sediment samples were incubated under methanogenic conditions for 94 days. Methane production rates were ≤0.5 nmol CH_4_ day^-1^ mL^-1^ and ≤0.6 nmol CH_4_ day^-1^ g^-1^, in the case of the water samples or sediments, respectively (**Figure 2**). Conversely, no methane increase was detected in cultures of samples from wells 5 and 9, and BES and sodium azide-amended cultures (data not shown).

All groundwater cultures exhibited methane production when simple methanogenic substrates were added (**Figure 2A**). Potential rates of CH_4_ production in acetate-amended groundwater cultures were between 0.1 and 2.87 nmol CH_4_ day^-1^ mL^-1^ (1.3–14 times higher than those of the non-amended controls). In methanol amended groundwater microcosms the rates were generally higher (increasing up to 44-fold compared to the non-amended controls), particularly in microcosms from wells 2, 3, 4, 6, and 8 (2.27–8.86 nmol CH_4_ day^-1^ mL^-1^). However, the highest methanogenic rates (up to 149 nmol CH_4_ day^-1^ mL^-1^) were detected in microcosms were H_2_ and CO_2_ had been added, increasing up to 1500 times in microcosms from well 10.

Acetate addition enhanced methane production (up to sevenfold) in microcosms with sediments 2 or 3 (4.6, and 1.1 nmol CH_4_ day^-1^ g^-1^ on average, respectively; **Figure 2B**). H_2_ and CO_2_ stimulated methane production only in microcosms with sediment 2. Among the hydrocarbon-amended microcosms, only those inoculated with sediments 1 or 2 and amended with *n*-hexadecane showed significantly increased methane production. The rates ranged from 8.7 ± 1.0 nmol CH_4_ day^-1^ mL^-1^ (sediment 2) to 16.4 ± 0.5 nmol CH_4_ day^-1^ mL^-1^ (sediment 1). The values for the non-inoculated cultures varied between 0.13 and 0.23 nmol CH_4_ day^-1^ mL^-1^.

Sulfate reduction rates varied between 1 and 60 nmol H_2_S produced mL^-1^ d^-1^ in the groundwater microcosms with no additional substrate. Lactate addition significantly accelerated sulfate reduction (up to 20-fold) in almost all groundwater cultures (rates from 0.4 to 0.6 μmol H_2_S mL^-1^ d^-1^). On the other hand, inocula from wells 3, 4, and 5 did not exhibit any sulfate reduction, even when lactate was added. Sulfate reduction in sediment cultures was only detectable in microcosms from sediment 3 amended with lactate (being 0.5 μmol H_2_S mL^-1^ d^-1^ on average).

### Quantification of Microbial Groups

The total number of cells in the groundwater samples, determined by direct count of SYBR Green-stained cells, was around 10^7^ cells mL^-1^ in samples 4, 5, and 10. Groundwater sample 3 showed the lowest numbers (10^4^ cells mL^-1^). Groundwater samples 1, 2, 6, 7, 8, and 9 had on the order of 10^6^ cells mL^-1^ (ranging from 2 to 9 × 10^6^).

**FIGURE 3:**
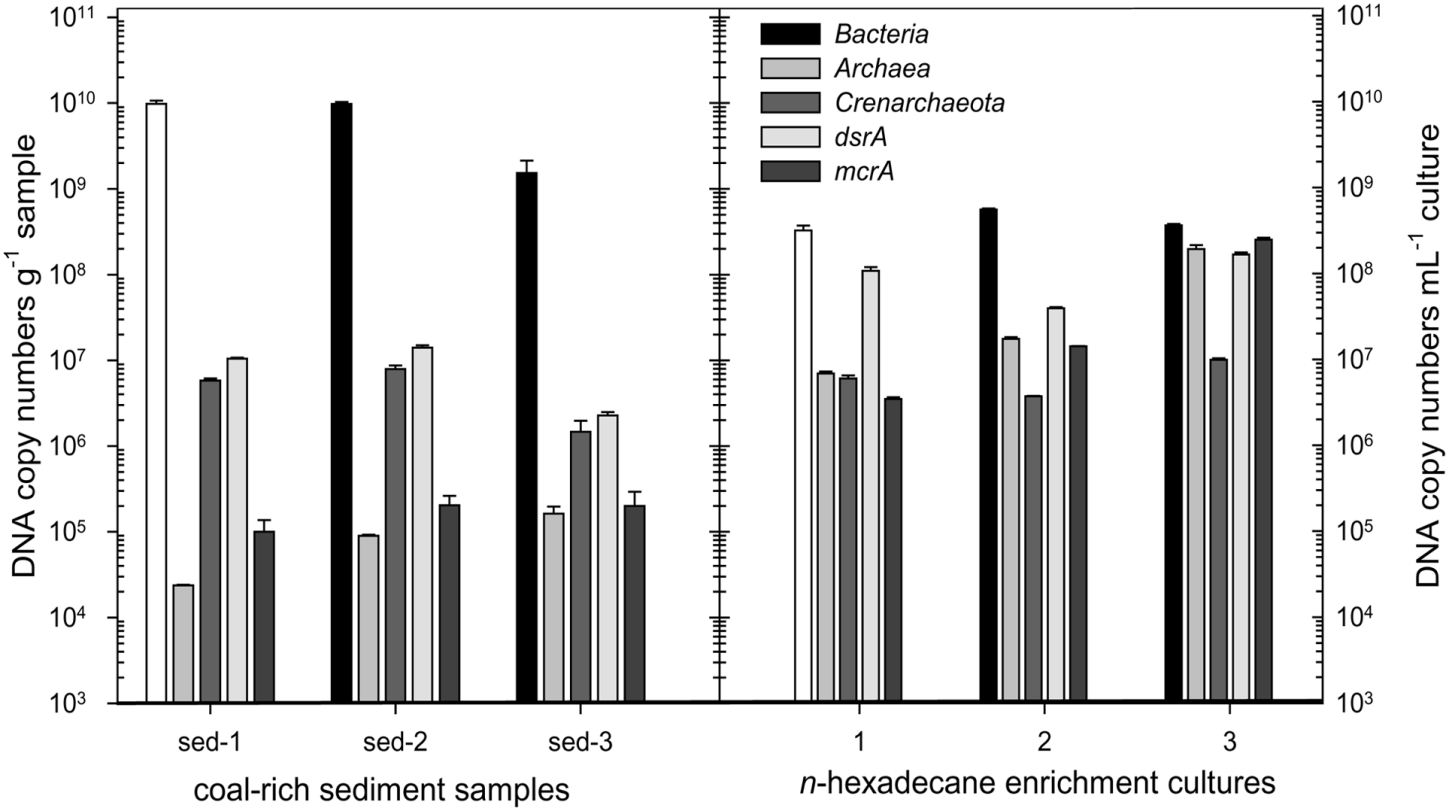
**Abundance of *Bacteria*, *Archaea*, *Crenarchaeota,* sulfate-reducing prokaryotes (*dsrA*) and methanogens (*mcrA*) in coal-rich sediment samples and their derived *n*-hexadecane-amended enrichment cultures, as determined by quantitative PCR.** The error bars represent the SD of three replicates.

The abundance of selected microbial groups in the coal-rich sediment samples was determined via quantitative (real time) PCR (Q-PCR; **Figure 3**). Average bacterial numbers ranged between 2 × 10^9^ and 1 × 10^10^ 16S rRNA gene copies g^-1^. However, archaeal numbers were much lower, in a range of 10^6^ copies g^-1^. Crenarchaeota were found in all three samples in nearly similar 16S rRNA gene copy numbers (6 × 10^6^, 8 × 10^6^, and 1 × 10^6^ copies g^-1^ in sediment sample 1, 2, and 3, respectively). However, only 10^4^–10^5^ 16S rRNA gene copies g^-1^ were retrieved using the primer set by Takai and Horikoshi (2000).

The quantitative detection of the dissimilatory sulfite reductase gene (*dsrA*) revealed sulfate-reducing prokaryotes in the range of 2 × 10^6^ (sediment 3) and 1 × 10^7^ copies g^-1^ in sediments 1 and 2. The abundance of methyl-coenzyme M reductase genes (*mcrA*) was between 1 and 2 × 10^5^ copies g^-1^.

The proportion of *Archaea*, methanogens and sulfate-reducers vs. *Bacteria* increased drastically in enrichment cultures amended with *n*-hexadecane, compared to the original sediments (**Figure 3**). Conversely, within the *Archaea*, the proportion of *Crenarchaeota* was lower in the *n*-hexadecane cultures.

### Phylogenetic Analysis of the Microbial Communities Composition

**FIGURE 4:**
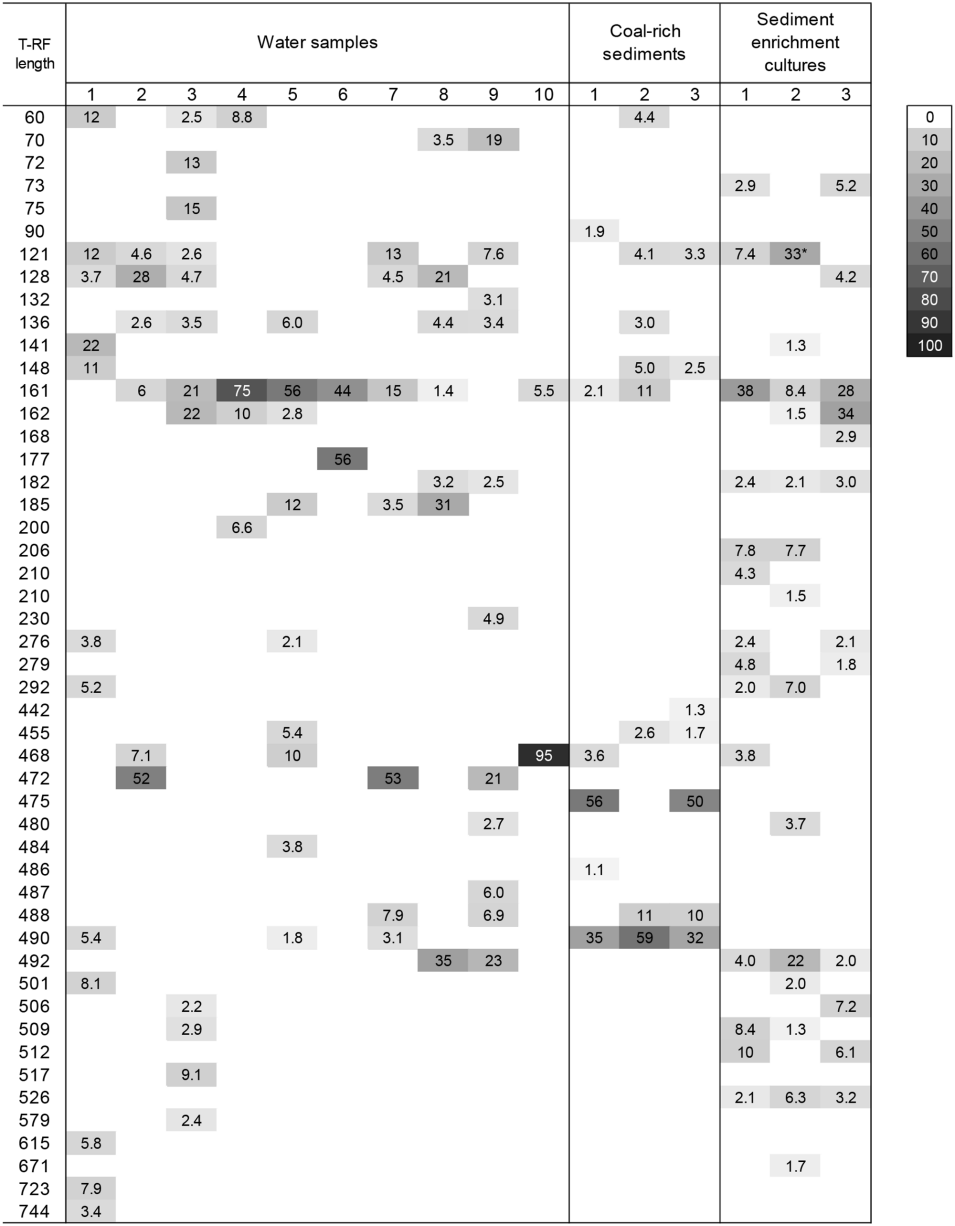
**Heatmap showing the T-RF found in Bacterial 16S rRNA gene T-RFLP fingerprints from different groundwater samples collected from deep aquifers of coal-rich sedimentary basin, coal-rich sediment samples, and the derived enrichment cultures amended with *n*-hexadecane.** The gray scale indicates the relative abundance of each fragment. Only values above 1% are given. *The abundance of this peak equals the maximum abundance for the sample.

**FIGURE 5:**
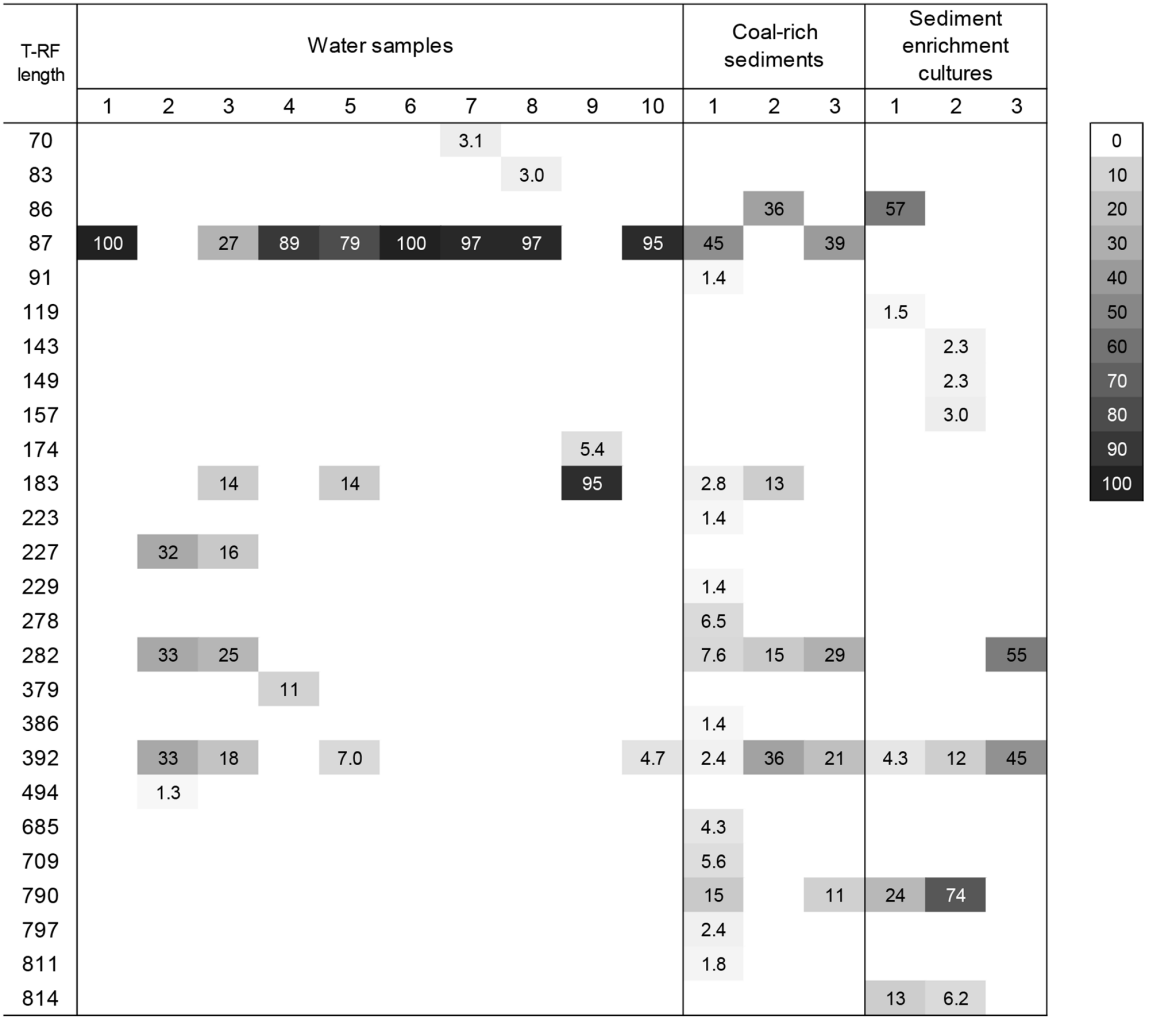
**Heatmap showing the T-RF found in Archaeal 16S rRNA gene T-RFLP fingerprints from different groundwater samples collected from deep aquifers of coal-rich sedimentary basin, coal-rich sediment samples, and the derived enrichment cultures amended with *n*-hexadecane.** The gray scale indicates the relative abundance of each fragment. Only values above 1% are given.

The diversity of the microbial communities in all environmental samples and *n*-hexadecane enrichment cultures was analyzed and compared using T-RFLP (**Figures 4** and **5**). Additionally, to identify the main fragments, clone libraries of the archaeal communities and pyrosequencing of the bacterial communities of selected sediment samples were performed, the results are shown in **Tables 2** and **3**, respectively. Because sediment sampling points were quite closely located, DNA for all three was pooled and subsequently analyzed.

**Table 2:** Phylogenetic affiliation of sequences produced by pyrosequencing analysis of bacterial 16S rRNA gene fragments retrieved from DNA extracted from unamended ligniteous coal-rich sediment and derived enrichment cultures (after 17 months) amended with *n*-hexadecane.

RDP Classifier with confidence threshold of 80%				T-RFs [bp]	Original sediment samples (pool)		Enrichment cultures			
							sed. 1		sed. 2	
Phylum	Class	Family	Main genus/genera		Reads	%	Reads	%	Reads.	%
Actinobacteria	Actinobacteria	Pseudonocardiaceae	*Pseudonocardia*		1	0.02	31	0.56	3	0.10
		Intrasporangiaceae	*Intrasporangium, Terrabacter*		3	0.06	103	1.86	2	0.07
		**Micrococcaceae**	***Arthrobacter, Micrococcus***	*161*	**2711**	**52.4**	56	1.01	24	0.78
		Streptomycetaceae	*Streptomyces*		2	0.04	32	0.58	7	0.23
		**Nocardioidaceae**	***Nocardioides, Marmoricola***		1	0.02	**215**	**3.87**	30	0.98
		Propionibacteriaceae	*Microlantus*		1	0.02	15	0.27	7	0.23
		Micromonosporaceae	*Dactylosporangium*		0	0	51	0.92	2	0.07
Bacteroidetes	Sphingobacteria	Chitinophagaceae			6	0.12	27	0.49	32	1.04
	Flavobacteria	Flavobacteriaceae	*Flavobacterium*		6	0.12	4	0.07	24	0.78
Proteobacteria	Epsilonproteobacteria	**Campylobacteraceae**	***Arcobacter, Sulfurospirillum***	*475, 468*	**434**	**8.38**	0	0	0	0
	Deltaproteobacteria	**Syntrophaceae**	***Desulfomonile, Smithella***		0	0	25	0.45	**136**	**4.44**
		Desulfovibrionaceae	*Desulfovibrio*		1	0.02	0	0	23	0.75
		Geobacteraceae		*168*	18	0.35	3	0.05	7	0.23
		**Desulfobulbaceae**	***Desulforhopalus***		1	0.02	**1618**	**29.1**	33	1.08
		**Desulfobacteraceae**	***Desulfatiferula***	*517*	0	0	21	0.38	**141**	**4.60**
	Betaproteobacteria	Burkholderiaceae	*Cupriavidus, Ralstonia*		6	0.12	77	1.39	2	0.07
		Comamonadaceae	*Acidovorax, Albidiferax, Polaromonas*	*488, 492*	**305**	**5.89**	19	0.34	5	0.16
		Oxalobacteraceae	*Hermiinimonas, Herbaspirillum, Massilia*	*141, 492*	**384**	**7.42**	16	0.29	21	0.69
		Hydrogenophilaceae	*Thiobacillus*		2	0.04	0	0	39	1.27
		**Methylophilaceae**	***Methylotenera***	*490, 492*	**119**	**2.30**	0	0	0	0
		Rhodocyclaceae	*Geofuchsia*	*488*	15	0.29	2	0.04	6	0.20
	Alphaproteobacteria	Bradyrhizobiaceae	*Bradyrhizobium*		4	0.08	30	0.54	19	0.62
		Phyllobacteriaceae	*Mesorhizobium*		1	0.02	12	0.22	12	0.39
		Hyphomicrobiaceae			2	0.04	13	0.23	28	0.91
		Rhodospirillaceae	*Oceanibaculum*		0	0	35	0.63	6	0.20
		Acetobacteraceae			0	0	25	0.45	0	0
	Gammaproteobacteria	Moraxellaceae	*Acinetobacter, Enhydrobacter*	*492*	29	0.56	7	0.13	0	0
		**Pseudomonadaceae**	***Pseudomonas***	*492*	**944**	**18.2**	23	0.41	13	0.42
		**Sinobacteraceae**	***Singularimonas, Sinobacter***		0	0	**379**	**6.83**	3	0.10
		Xanthomonadaceae	*Lysobacter*		0	0	32	0.58	15	0.49
Gemmatimonadetes	Gemmatimonales	Gemmatimonadaceae	*Gemmatimonas*		1	0.02	24	0.43	20	0.65
Nitrospirae	Nitrospira	Nitrospiraceae	*Nitrospira*		0	0	8	0.14	29	0.95
Firmicutes	Clostridia	Clostridiaceae 1	*Clostridium* sensu stricto		0	0	22	0.40	18	0.59
		Eubacteriaceae	*Acetobacterium*		11	0.21	0	0	0	0
	Bacilli	**Bacillaceae 1**	***Bacillus***		0	0	38	0.68	**73**	**2.38**
		Paenibacillaceae 1	*Paenibacillus*		0	0	24	0.43	31	1.01
		Planococcaceae			0	0	3	0.05	36	1.17
Planctomycetes	Planctomycetacia	Planctomycetaceae	*Singulisphaera*		0	0	**114**	**2.05**	**23**	**0.75**
Chloroflexi	Anaerolineae	**Anaerolineaceae**			0	0	**242**	**4.36**	**87**	**2.84**
Miscellaneous					30	0.58	299	5.39	188	6.14
Unclassified					138	2.67	1907	34.3	1919	62.6
**Total**					5176	100	5552	100	3064	100

A total of 5176 sequences (coal-rich sediments), 5552 sequences (enrichment culture 1), and 3064 sequences (enrichment cultures 2) were included in this calculation. Numbers in cursive characters show accordance with dominant peaks in the T-RFLP fingerprint plots (**Figure 4**). Values in bold represent taxa to which at least 2% of the reads have been assigned in at least one of the samples.

**Table 3:** Phylogenetic affiliation of clone sequences produced via clone libraries of archaeal 16S rRNA gene fragments retrieved from DNA extracted from unamended ligniteous coal-rich sediment and derived enrichment cultures (after 17 months of incubation) amended with *n*-hexadecane.

RDP Classifier with confidence threshold of 80%				T-RFs [bp]	Original sediment samples (pool)		Enrichment cultures					
							Sediment 1		Sediment 2		Sediment 3	
Phylum	Class	Order	Main genus/genera		No of sequence	%	No of sequence	%	No of sequence	%	No of sequence	%
Euryarchaeota	Methanomicrobia	Methanosarcinales	*Methanosaeta*	*282*	1	1	-	-	-	-	-	-
			*Methanosarcina*	*183*	1	1	-	-	-	-	-	-
			*Methanomethylovorans*	*790, 831*	1	1	-	-	2	2	-	-
			Unclassified		22	23	-	-	-	-	-	-
		Methanomicrobiales	*Methanoregula*	*392*			-	-	-	-	2	2
		Unclassified			3	3	-	-	-	-	-	-
Crenarchaeota	Thermoprotei	Unclassified			44	46	87	95	92	97	37	39
Unclassified					24	25	5	5	1	1	56	59
**Total**					96	100	92	100	95	100	95	100

A total of 96 sequences (coal-rich sediments), 92 sequences (enrichment culture 1) and 95 sequences (enrichment cultures 2 and 3) were included in this calculation. Numbers in cursive characters show accordance with dominant peaks in the T-RFLP fingerprint plots (**Figure 5**).

#### Bacterial Communities

Terminal restriction fragment length polymorphism profiles of the three different coal-rich sediment samples exhibited only few T-RF (**Figure 4**), reflecting the predominance of a small number of taxa. The most significant peaks were 476-bp T-RF, corresponding to the epsilonproteobacterium *Arcobacter*; 488-bp T-RF, belonging to *Acidovorax*, and 492-bp T-RF, which matched with *Pseudomonas* sp. and *Acinetobacter* (*Pseudomonadales*), and several *Betaproteobacteria* affiliated to *Oxalobacteraceae*, *Comamonadaceae* and *Methylophilaceae*. Minor peaks corresponded to the genera *Sulfurospirillum* (468-bp T-RF) and *Arthrobacter* sp. (161-bp T-RF).

5176 sequences were retrieved from the pyrosequencing analysis of sediment samples. The majority of these pyrosequences corresponded to representatives of *Actinobacteria* (52.5%), mostly *Arthrobacter* (52.4%); *Gammaproteobacteria* (18.8%), most of them belonging to *Pseudomonas* (18.2%); *Betaproteobacteria* (16.1%), represented by members of the *Oxalobacteraceae* (like *Massilia*), *Comamonadaceae* (mainly of genera *Acidivorax* or *Albidiferax*, 5.9%) and *Methylophilaceae* (e.g., genus *Methylotenera*, 2.3%) families; *Epsilonproteobacteria* (8.4%), mainly *Arcobacter* spp. (7.4%) and *Sulfurospirillum*.

According to the TRFL-P profiles, bacterial communities from *n*-hexadecane amended sediment-derived cultures (**Figure 3**) were more diverse (i.e., presented several predominant peaks), which was also confirmed by pyrosequencing (**Table 2**), and more similar to groundwater rather than coal-sediment samples. Most of the identified phylotypes belonged to *Proteobacteria*, followed by members of *Actinobacteria, Anaerolineae,* and *Clostridia*. In addition, there was a shift of the bacterial dominance toward the *Deltaproteobacteria*, mainly *Desulfobacterales* (like *Desulforhopalus* or *Desulfatiferula*), *Desulfuromonadales* (belonging to *Geobacteraceae*) and Syntrophobacterales (*Desulfomonile* and *Smithella*), and the *Anaerolineae*. The remaining sequences fell in the classes of *Alphaproteobacteria*, *Betaproteobacteria*, *Gammaproteobacteria*, *Clostridia* (e.g., *Bacillus* and *Paenibacillus*) and Actinobacteria (e.g., *Arthrobacter, Micrococcus, Nocardioides* and *Marmoricola* **Table 2**).

T-RFLP patterns of groundwater samples were highly variable, but most of them presented only a few predominant peaks (**Figure 4**). Dominant T-RFs were coincident to those from sediment samples: 161-bp T-RF (*Arthrobacter)* 468-bp T-RF (*Sulfurospirillum*), 475-bp T-RF (*Arcobacter*), and 492-bp T-RF (assigned to several Gammaproteobacteria and *Betaproteobacteria*, as already stated). Another genus abundant in well 3, was *Desulfatiferula* (*Deltaproteobacteria*)*,* corresponding to the 517-bp T-RF. In addition the community presented different fragments, which could not be identified (**Figure 4**).

#### Archaeal Communities

The phylogenetic analysis of archaeal 16S rRNA gene fragments (**Table 3**; **Figure 4**) confirmed the presence of methanogenic *Archaea* in the coal-rich sediment samples, mainly *Methanosaeta* sp. (24%), but also *Methanosarcina* (1%; 183-bp T-RF) and *Methanomethylovorans* (1%; 790-bp T-RF). Furthermore, 46% of sequences were affiliated to unclassified *Thermoprotei* within the phylum *Crenarchaeota*.

The majority of the generated sequences from enrichment cultures belonged to unclassified *Thermoprotei* (**Table 3**; **Figure 5**) or methanogenic *Archaea*, like *Methanomethylovorans* (790-bp T-RF, abundant in enrichment culture 2) or phylotypes closely related to *Methanosaeta concilii* (282-bp T-RF) and *Methanoregula formicicum* (392-bp T-RF). Archaeal community diversity was lower than bacterial diversity in all groundwater samples. Only the T-RFLP fingerprints of wells 2 and 3 presented more than one T-RF peak (**Figure 5**), corresponding to genera *Methanosarcina* (183-bp T-RFs), *Methanosaeta* (282-bp T-RFs), and *Methanoregula* (392-bp T-RFs). Most of the 16S rRNA gene sequences were related to members of the class *Methanomicrobia* (*Euryarchaeota*) and unclassified *Thermoprotei*. In addition, two dominant peaks (227-bp T-RF, present in wells 2 and 3, and 87-bp T-RF could not be assigned.

## Discussion

Groundwater and coal-rich sediment samples were investigated to assess the ability of subsurface microbial communities to produce methane from coal-related hydrocarbons. Both water and sediments showed evidence for biological activity, particularly for methanogenic processes, as reflected in the high concentrations of DOC and on the biogenic isotopic signatures of methane and CO_2_. This activity was consistent with the presence of sufficient amounts of N and P, trace elements and electron acceptors to support microbial growth and potentially stimulate the initial steps of hydrocarbon degradation, which could eventually lead to methane production.

### Putative Hydrocarbon Degrading Microorganisms in Lignite-Bearing Sediments and Associated Waters

To identify the microorganisms responsible for the degradation of complex organic matter of ligniteous coals to substrates for methanogenesis, microbial communities from groundwater and sediment samples were studied using T-RFLP fingerprinting combined with pyrosequencing of 16S rRNA gene fragments.

Microbial communities in groundwater and coal-rich sediments were dominated by putative hydrocarbon-degrading taxa affiliated to *Actinobacteria* (such as *Arthrobacter*) and *Proteobacteria*, mostly *Gammaproteobacteria* belonging to *Pseudomonadales*, and *Betaproteobacteria*, including *Acidovorax*, *Burkholderia*, *Polaromonas*, *Geofuchsia,* and *Ralstonia* (**Figure 4**; **Table 2**). Many of these have been frequently found in other coalbed reservoirs (Shimizu et al., 2007; Li et al., 2008; Penner et al., 2010), associated waters (Lemay and Konhauser, 2006; Midgley et al., 2010; Penner et al., 2010), and other hydrocarbon-rich environments like petroleum reservoirs (Li et al., 2007; Tang et al., 2012) or petroleum-contaminated soils or sediments (Reisfeld et al., 1972), and are known to degrade a variety of hydrocarbon and related substituted molecules (Parales, 2010).

Within the hydrocarbon degrading microorganisms were nitrate reducers related to *Pseudomonas, Acinetobacter, Arcobacter, Arthrobacter,* and *Geofuchsia.* This was consistent with the nitrate content, the absence of oxygen and with the close association of groundwater to coal-rich sediments, where different hydrocarbons are available as substrate for microbes. *Pseudomonas* species are often capable to degrade alkane, alkene- and polycyclic aromatic hydrocarbons (Rockne et al., 2000). *Acinetobacter* strains have been shown to be able to efficiently degrade short- and long-chain linear alkanes, isoalkanes and various aromatic compounds (Mbadinga et al., 2011; Li et al., 2012). *Arthrobacter* species are gram-negative soil *Actinomycetes*, able to degrade cellulose and other polysaccharides (Li et al., 2008), as well as polycyclic aromatic hydrocarbons (Kallimanis et al., 2009). *Arthrobacter* sp. may play an important role in biodegradation of coal-associated hydrocarbon compounds at the coal-water boundary layer within the sites investigated here. *Georgfuchsia toluolica* degrades BTEX compounds using nitrate, Fe(III) or Mn(IV) as electron acceptor (Weelink et al., 2009).

Hydrocarbon-degrading sulfate reducers were identified as well. For example, *Desulfatiferula* sp. was exclusively found in groundwater sample 3 where the sulfate concentration was higher than in the other groundwater samples. *Desulfatiferula olefinivorans*, a mesophilic sulfate-reducing bacterium isolated from oil-polluted sediment, exclusively oxidizes long-chain alkenes and fatty acids incompletely to acetate, using only sulfate as electron acceptor (Cravo-Laureau et al., 2007).

All these hydrocarbon-degrading microorganisms may be involved in the initial breakdown of coal and its transformation into more labile compounds which might be further used by the reminder of the microbial community. In this respect, *Pseudomonadales* are considered to play an important role in the degradation of organic matter of lignite and sub-bituminous coals (Machnikowska et al., 2002; Jones et al., 2010).

Other taxa, such as the genera *Acidovorax*, *Arcobacter,* or *Methylotenera,* have been previously found in petroleum and coal-related environments (Voordouw et al., 1996; Li et al., 2008; Midgley et al., 2010; Penner et al., 2010; Liu and Liu, 2013). So they might also be related to hydrocarbon biodegradation. *Methylotenera* spp., for example, utilizes fermentation products (Kalyuzhnaya et al., 2006).

The identification of aerobic or facultative denitrifying together with sulfate- and iron-reducing hydrocarbon-degrading microorganisms suggests a high metabolic versatility of the microbial community, which thus could easily respond to changes in the availability of electron acceptors, and continue to degrade coal regardless of the electron acceptor available. The presence of relatives of the *Sulfurospirillum* spp. and *Arcobacter* spp. points to an important role of sulfur and nitrogen cycling. Some *Arcobacter* species are able to reduce nitrate and oxidize sulfide, while *Sulfurospirillum* species have extremely diverse metabolic features and possibly reduce sulfur as well as oxidize nitrite (Tang et al., 2012).

Interestingly, a high abundance of 16S rRNA gene sequences affiliated to the unclassified *Thermoprotei* (within the phylum Crenarchaeota) was found in all coal-rich sediment samples. Kemnitz et al. (2007) showed that *Crenarchaeota* are ubiquitous and commonly found in low temperature environments. However, little is known about their physiological characteristics and their ecological importance, because the majority of this group of microorganisms has not been cultivated yet. Nevertheless, recently, relatives of hyperthermophilic *Thermoprotei* were detected in production waters from high-temperature petroleum reservoirs in China (e.g., Li et al., 2007; Ren et al., 2011; Tang et al., 2012), which suggests a capability of hydrocarbon degradation or a close contribution to the degradation process.

The microbial community identified in the groundwater samples contains a broad spectrum of putative hydrocarbon degraders, able to degrade complex organic compounds such as long-chain alkenes, polycyclic aromatic hydrocarbons, BTEX, and other coal-associated compounds (e.g., fatty acids) to lower molecular weight compounds which are utilizable by methanogens.

### Hydrocarbon and Low Molecular Weight Organic Acid Degradation in Enrichment Cultures

To demonstrate microbial growth and conversion of coal-rich sediments or hydrocarbons to methane and carbon dioxide by coal-associated communities, enrichment cultures amended with hydrocarbon substrates or original coal as sole carbon and energy source were investigated. According to Orem et al. (2010), alkanes are among the primary intermediates in the biodegradation pathway from coal-derived geopolymers. We assume that the enriched microorganisms directly involved in this process, with different degradation steps performed by specifically adapted microbial groups, are also active in their native coal-associated environments.

Microbial communities from *n*-hexadecane enrichment cultures consisted predominantly of deltaproteobacteria syntrophic and sulfate-reducing taxa belonging to *Desulfobacterales, Desulfuromonadales, Desulfovibrionales,* and *Syntrophobacterales* known for their ability to degrade long-chain fatty acids (Sousa et al., 2009), correlating well with the detection of high amounts of *dsrA* genes via Q-PCR. In a previous study we observed that the addition of electron acceptors such as manganese, ferric iron or low concentrations of sulfate (below 5 mM), can accelerate *n*-hexadecane-dependent methanogenesis (Siegert et al., 2011). On the other hand, *Thermoprotei* were highly enriched in these *n*-hexadecane-amended cultures, which reinforces the hypothesis of their contribution in the biodegradation of hydrocarbons.

In the present study, *n*-hexadecane proved to be a good methanogenic substrate for the lignite-rich sediment-associated microbial community. However, it did not stimulate methanogenesis in groundwater inoculated microcosms, unlike acetate, methanol or CO_2_ and H_2_. Therefore, it seems likely that there is an inflow of low-molecular-weight organic acids (LMWOA) and other fermentation products from the underlying lignite-rich sediments into the coal-associated aquifer.

Vieth et al. (2008) demonstrated that some LMWOA, such as acetate, formate, and oxalate, could be released from low rank ligniteous coals into surrounding water, providing a carbon source for the associated microbiota. This process depends on the organic matter and maturity of the coal material. Their study also showed that the water soluble acids were transported by diffusion and/or advection from the coal layers to the adjacent carrier lithologies to support the deep biosphere (Vieth et al., 2008). According to Bergmann (1999) lignite seams are the main source of organic carbon in the aquifers. Available LMWOA in subsurface aquatic systems have the potential for providing a sufficient energy source for microbial life, especially for methanogenic activity (Vieth et al., 2008). Moreover, LMWOA could also be produced by microbial degradation of organic carbon material from low rank coals (Laborda et al., 1997; Fakoussa and Hofrichter, 1999). Further, several studies demonstrated that the natural microbial activity was stimulated at lignite-rich/sediment interfaces (Ulrich et al., 1998) and at the interface between aquifer and lignite seam (Detmers et al., 2001). Both studies concluded that microbial fermentation of organic matter in lignite-rich sediments provides LMWOA, which in turn feed the sulfate-reducing bacteria.

Because of the high similarity of the microbial communities in both geosystems, we suppose that not only LMWOA but also the indigenous microbes of coal seam layers were leached out into the groundwater and thus affected the microbial groundwater community.

### CO_2_ Reduction as Prevailing Methanogenic Pathway in Groundwater

Methanogenic *Archaea* were predominant in both groundwater and sediment samples, which was consistent with the occurrence of biogenic methane. In both cases, the methanogenic community included putative acetoclastic, hydrogenotrophic and methylotrophic methanogens closely related to *Methanosarcina horonobensis*, *Methanosaeta concilii,* and *Methanoregula formicicum.* Sediment samples contained *Methanomethylovorans* sp. as well.

Related taxa had previously been found in other coal-related locations, such as coalbed wells from the Fort Union Formation in the Powder River Basin (Green et al., 2008) or a deep coal seam in northern Japan (Shimizu et al., 2007), and on methanogenic enrichment cultures of samples from abandoned coal mines (Beckmann et al., 2011b).

*Methanoregula formicicum*, detected in nearly all groundwater samples, is able to use formate or H_2_ and CO_2_ as substrates for growth (Yashiro et al., 2011).

Relatives of *Methanomethylovorans*, described by Lomans et al. (1999) and Jiang et al. (2005), utilize methanol, methylated amines, dimethyl sulfide and methane-thiol for methanogenic activity, and have been found in eutrophic fresh water sediments (Lomans et al., 1999), rice field soils (Lueders et al., 2001) and oil contaminated groundwater (Watanabe et al., 2002)

*Methanosarcina* sp. and *Methanosaeta* sp. are the only two known genera which can utilize acetate as a substrate for methanogenesis. The genus *Methanosaeta* has traditionally been considered to exclusively use acetate as sole source for energy or as substrate for methanogenesis (Patel and Sprott, 1990), as it is unable to use H_2_ or formate as electron donors for methane production from CO_2_. However, in a recent study, Rotaru et al. (2014) demonstrated that at least some *Methanosaeta* species are capable of accepting electrons from other microorganisms for CO_2_ reduction. In this respect, the carbon isotopic composition (δ^13^C) of methane and CO_2_ (**Figure 1**) indicated a dominance of methanogenic CO_2_ reduction in most of the groundwater wells, including 2 and 3, suggesting that *Methanosaeta* could also take part in this methanogenic process.

*Methanosarcina* sp. dominated in samples from well 9, where according to its isotopic composition methane could have a mixed or acetoclastic origin. *Methanosarcina horonobensis*, isolated from deep subsurface groundwater of a mudstone formation, is able to metabolize methanol, methylated compounds (e.g., di- and trimethylamine, dimethylsulfide) or acetate as sole energy source (Shimizu et al., 2011). Mixed or acetoclastic methane was also observed in samples 7 and 10 and in other coal seams characterized by the movement of meteoric water containing nutrients and microbiota (e.g., Flores et al., 2008; Klein et al., 2008). Nevertheless, these samples exhibited a higher methane production potential in enrichment cultures where a mixture of H_2_ and CO_2_ was added. Conversely, the sediment enrichment cultures predominantly produced methane from acetate. H_2_ and CO_2_ did either not enhance methanogenesis or the effect was lower, paralleling previous results by Krüger et al. (2008) and Green et al. (2008). Krüger et al. (2008) suggested that acetate would be a central intermediate in the transformation of coal to methane. In summary, the methanogens found in our study seemed to be adapted to their local environmental conditions.

Methanogenic archaeal communities (including *Methanomethylovorans* sp. and phylotypes closely related to *Methanosaeta concilii* and *Methanoregula formicicum*) together with their syntrophic bacterial partners and unclassified *Crenarchaeota* were successfully enriched in cultures amended with *n*-hexadecane as sole source of carbon and energy, as reflected in the increase of the proportion of the archaeal, crenarchaeal, methanogenic, and sulfate-reducing populations (**Figure 3**). These enrichments were able to produce methane, which proves that microbial communities inhabiting the coal-rich sediments can convert *n*-hexadecane, an intermediate of coal biodegradation (Orem et al., 2010), to methane. The predominance of one or another methanogenic pathway must be site-dependent and can be influenced by a variety of factors, such as temperature, changes on the availability of electron acceptors, etc. leading to the formation of a highly adapted microbial community, in which acetoclastic as well as hydrogenotrophic methanogens are abundant.

## Conclusions

The investigated coal-rich sediments in contact with groundwater provide an important source of organic material and electron acceptors for microbial life in the subsurface biosphere. This organic material possibly includes low-molecular-weight organic acids derived from coal biodegradation. They were then partly transferred into the coal-associated aquifer system by circulating natural meteoric waters. The detected fermenting and sulfate-reducing bacterial consortia are able to degrade complex organic material from coal to lower molecular weight compounds which consequently directly influence the microbial interactions in the groundwater system. The resulting end products, like H_2_ or small organic acids, are potential substrates for methanogenic archaea. The identified methanogenic archaea point to the simultaneous occurrence of both methanogenic pathways, although hydrogenotrophic methanogenesis seemed to dominate in groundwaters. This assumption was supported by δ^13^C_CH4_- and δD_CH4_-values, of a respective microbial origin, in coal-rich sediment and groundwater samples.

## Conflict of Interest Statement

The authors declare that the research was conducted in the absence of any commercial or financial relationships that could be construed as a potential conflict of interest.

## Footnotes

1 http://www.microsynth.ch/

2 http://www.geneious.com/

3 http://decipher.cee.wisc.edu/FindChimeras.html

4 http://blast.ncbi.nlm.nih.gov/Blast.cgi

5 http://rdp.cme.msu.edu/classifier/classifier.jsp

6 http://www.arb-silva.de/aligner/

7 http://www.mothur.org/

## References

[B1] Altschul S. F. Gish W. Miller W. Myers E. W. Lipman D. J. (1990). Basic local alignment search tool. *J. Mol. Biol.* 215 403–410 10.1016/S0022-2836(05)80360-2 2231712

[B2] Beckmann S. Krüger M. Engelen B. Gorbushina A. A. Cypionka H. (2011a). Role of bacteria, archaea and fungi involved in methane release in abandoned coal mines. *Geomicrobiol. J.* 28 347–358 10.1080/01490451.2010.503258

[B3] Beckmann S. Lueders T. Krüger M. Von Netzer F. Engelen B. Cypionka H. (2011b). Acetogens and acetoclastic methanosarcinales govern methane formation in abandoned coal mines. *Appl. Environ. Microbiol.* 77 3749–3756 10.1128/AEM.02818-10 21460109 PMC3127600

[B4] Bergmann A. (1999). “Hydrogeochemische Untersuchungen anoxischer Redox-Prozesse in tiefen Porengrundwasserleitern der Niederrheinischen Bucht,” im *Umfeld des Tagebaus Garzweiler I, Bochumer Geologische und Geotechnische Arbeiten 51* (Bochum: Institut für Geologie, Bochum Ruhr-University) 167

[B5] Cord-Ruwisch R. (1985). A quick method for the determination of dissolved and precipitated sulfides in cultures of sulfate-reducing bacteria. *J. Microbiol. Meth.* 4 33–36 10.1016/0167-7012(85)90005-3

[B6] Cravo-Laureau C. Labat C. Joulian C. Matheron R. Hirschler-Réa A. (2007). Desulfatiferula olefinivorans gen. nov., sp. nov., a long-chain n-alkene-degrading, sulfate-reducing bacterium. *Int. J. Syst. Evol. Microbiol.* 57 2699–2702 10.1099/ijs.0.65240-0 17978243

[B7] Culman S. W. Bukowski R. Gauch H. G. Cadillo-Quiroz H. Buckley D. H. (2009). T-rex: Software for the processing and analysis of t-rflp data. *BMC Bioinformatics* 10:171 10.1186/1471-2105-10-171 PMC2702334 19500385

[B8] Dekov V. M. Kamenov G. D. Savelli C. Stummeyer J. Marchig V. (2007). Origin of basal dolomitic claystone in the marsili basin, tyrrhenian sea. *Mar. Geol.* 236 121–141 10.1016/j.margeo.2006.10.021

[B9] DeLong E. F. (1992). Archaea in coastal marine environments. *PNAS* 89 5685–5689 10.1073/pnas.89.12.5685 1608980 PMC49357

[B10] DeSantis T. Z. Hugenholtz P. Larsen N. Rojas M. Brodie E. L. Keller K. (2006). Greengenes, a chimera-checked 16S rRNA gene database and workbench compatible with ARB. *Appl. Environ. Microbiol.* 72 5069–5072 10.1128/AEM.03006-05 16820507 PMC1489311

[B11] Detmers J. Schulte U. Strauss H. Kuever J. (2001). Sulfate reduction at a lignite seam: microbial abundance and activity. *Microb. Ecol.* 42 238–247 10.1007/s00248-001-1014-8 12024249

[B12] Fakoussa R. M. Hofrichter M. (1999). Biotechnology and microbiology of coal degradation. *Appl. Microbiol. Biotechnol.* 52 25–40 10.1007/s002530051483 10461367

[B13] Feisthauer S. Siegert M. Seidel M. Richnow H. H. Zengler K. Gründger F. (2010). Isotopic fingerprinting of methane and co2 formation from aliphatic and aromatic hydrocarbons. *Org. Geochem.* 41 482–490 10.1016/j.orggeochem.2010.01.003

[B14] Flores R. M. Rice C. A. Stricker G. D. Warden A. Ellis M. S. (2008). Methanogenic pathways of coal-bed gas in the powder river basin, united states: the geologic factor. *Int. J. Coal Geol.* 76 52–75 10.1016/j.coal.2008.02.005

[B15] Fry J. C. Horsfield B. Sykes R. Cragg B. A. Heywood C. Kim G. T. (2009). Prokaryotic populations and activities in an interbedded coal deposit, including a previously deeply buried section (1.6*–*2.3 km) above ∼ 150 ma basement rock. *Geomicrobiol. J.* 26 163–178 10.1080/01490450902724832

[B16] Green M. S. Flanegan K. C. Gilcrease P. C. (2008). Characterization of a methanogenic consortium enriched from a coalbed methane well in the Powder River Basin, U.S.A. *Int. J. Coal Geol.* 76 34–45 10.1016/j.coal.2008.05.001

[B17] Guo H. Liu R. Yu Z. Zhang H. Yun J. Li Y. (2012). Pyrosequencing reveals the dominance of methylotrophic methanogenesis in a coal bed methane reservoir associated with eastern ordos basin in china. *Int. J. Coal Geol.* 93 56–61 10.1016/j.coal.2012.01.014

[B18] Herrmann S. Kleinsteuber S. Chatzinotas A. Kuppardt S. Lueders T. Richnow H.-H. (2010). Functional characterization of an anaerobic benzene-degrading enrichment culture by DNA stable isotope probing. *Environ. Microbiol.* 12 401–411 10.1111/j.1462-2920.2009.02077.x 19840104

[B19] Jiang B. Parshina S. N. Van Doesburg W. Lomans B. P. Stams A. J. M. (2005). Methanomethylovorans thermophila sp. nov., a thermophilic, methylotrophic methanogen from an anaerobic reactor fed with methanol. *Int. J. Syst. Evol. Microbiol.* 55 2465–2470 10.1099/ijs.0.63818-0 16280511

[B20] Jones E. J. P. Voytek M. A. Corum M. D. Orem W. H. (2010). Stimulation of methane generation from nonproductive coal by addition of nutrients or a microbial consortium. *Appl. Environ. Microbiol.* 76 7013–7022 10.1128/AEM.00728-10 20817801 PMC2976240

[B21] Kallimanis A. Kavakiotis K. Perisynakis A. Spröer C. Pukall R. Drainas C. (2009). Arthrobacter phenanthrenivorans sp. nov., to accommodate the phenanthrene-degrading bacterium arthrobacter sp. Strain sphe3. *Int. J. Syst. Evol. Microbiol.* 59 275–279 10.1099/ijs.0.000984-0 19196765

[B22] Kalyuzhnaya M. G. Bowerman S. Lara J. C. Lidstrom M. E. Chistoserdova L. (2006). Methylotenera mobilis gen. nov., sp. nov., an obligately methylamine-utilizing bacterium within the family methylophilaceae. *Int. J. Syst. Evol. Micr.* 56 2819–2823 10.1099/ijs.0.64191-0 17158982

[B23] Kemnitz D. Kolb S. Conrad R. (2007). High abundance of crenarchaeota in a temperate acidic forest soil. *FEMS Microbiol. Ecol.* 60 442–448 10.1111/j.1574-6941.2007.00310.x 17391330

[B24] Klein D. A. Flores R. M. Venot C. Gabbert K. Schmidt R. Stricker G. D. (2008). Molecular sequences derived from paleocene fort union formation coals vs. associated produced waters: implications for cbm regeneration. *Int. J. Coal Geol.* 76 3–13 10.1016/j.coal.2008.05.023

[B25] Krüger M. Beckmann S. Engelen B. Thielemann T. Cramer B. Schippers A. (2008). Microbial methane formation from hard coal and timber in an abandoned coal mine. *Geomicrobiol. J.* 25 315–321 10.1080/01490450802258402

[B26] Krüger M. Frenzel P. Conrad R. (2001). Microbial processes influencing methane emission from rice fields. *Glob. Chang. Biol.* 7 49–63 10.1046/j.1365-2486.2001.00395.x

[B27] Laborda F. Fernández M. Luna N. Monistrol I. F. (1997). Study of the mechanisms by which microorganisms solubilize and/or liquefy spanish coals. *Fuel Process. Technol.* 52 95–107 10.1016/S0378-3820(97)00019-2

[B28] Lane D. J. (1991). “16s/23s rRNA sequencing,” in *Nucleic Acid Techniques in Bacterial Systematics* eds Stackebrandt E. Goodfellow M. (New York, NY: John Wiley & Sons) 115–175.

[B29] Lemay T. G. Konhauser K. O. (2006). *Water* *Chemistry of Coalbed Methane Reservoirs*. Alberta Energy and Utilities Board, EUB/AGS Special Report, Edmonton, AB.

[B30] Li D. Hendry P. Faiz M. (2008). A survey of the microbial populations in some australian coalbed methane reservoirs. *Int. J. Coal Geol.* 76 14–24 10.1016/j.coal.2008.04.007

[B31] Li H. Yang S.-Z. Mu B.-Z. Rong Z.-F. Zhang J. (2007). Molecular phylogenetic diversity of the microbial community associated with a high-temperature petroleum reservoir at an offshore oilfield. *FEMS Microbiol. Ecol.* 60 74–84 10.1111/j.1574-6941.2006.00266.x 17286581

[B32] Li W. Wang L.-Y. Duan R.-Y. Liu J.-F. Gu J.-D. Mu B.-Z. (2012). Microbial community characteristics of petroleum reservoir production water amended with n-alkanes and incubated under nitrate-, sulfate-reducing and methanogenic conditions. *Int. Biodeterior. Biodegrad.* 69 87–96 10.1016/j.ibiod.2012.01.005

[B33] Liu Z. Liu J. (2013). Evaluating bacterial community structures in oil collected from the sea surface and sediment in the northern Gulf of Mexico after the *Deepwater Horizon* oil spill. *Microbiologyopen* 2 492–504 10.1002/mbo3.89 23568850 PMC3684762

[B34] Lomans B. P. Maas R. Luderer R. Op Den Camp H. J. M. Pol A. Van Der Drift C. (1999). Isolation and characterization of *Methanomethylovorans hollandica* gen. nov., sp. nov., isolated from freshwater sediment, a methylotrophic methanogen able to grow on dimethyl sulfide and methanethiol. *Appl. Environ. Microbiol.* 65 3641–3650. 10427061 10.1128/aem.65.8.3641-3650.1999 PMC91546

[B35] Lueders T. Chin K.-J. Conrad R. Friedrich M. (2001). Molecular analyses of methyl-coenzyme m reductase α-subunit (mcra) genes in rice field soil and enrichment cultures reveal the methanogenic phenotype of a novel archaeal lineage. *Environ. Microbiol.* 3 194–204 10.1046/j.1462-2920.2001.00179.x 11321536

[B36] Lueders T. Manefield M. Friedrich M. W. (2004). Enhanced sensitivity of DNA- and rrna-based stable isotope probing by fractionation and quantitative analysis of isopycnic centrifugation gradients. *Environ. Microbiol.* 6 73–78 10.1046/j.1462-2920.2003.00536.x 14686943

[B37] Machnikowska H. Pawelec K. Podgórska A. (2002). Microbial degradation of low rank coals. *Fuel Process. Technol.* 77–78, 17–23 10.1016/S0378-3820(02)00064-4

[B38] Mbadinga S. M. Wang L.-Y. Zhou L. Liu J.-F. Gu J.-D. Mu B.-Z. (2011). Microbial communities involved in anaerobic degradation of alkanes. *Int. Biodeterior. Biodegrad.* 65 1–13 10.1016/j.ibiod.2010.11.009

[B39] Midgley D. J. Hendry P. Pinetown K. L. Fuentes D. Gong S. Mitchell D. L. (2010). Characterisation of a microbial community associated with a deep, coal seam methane reservoir in the gippsland basin, australia. *Int. J. Coal Geol.* 82 232–239 10.1016/j.coal.2010.01.009

[B40] Nadkarni M. A. Martin F. E. Jacques N. A. Hunter N. (2002). Determination of bacterial load by real-time pcr using a broad-range (universal) probe and primers set. *Microbiology* 148 257–266. 11782518 10.1099/00221287-148-1-257

[B41] Ochsenreiter T. Selezi D. Quaiser A. Bronch-Osmolovskaya L. Schleper C. (2003). Diversity and abundance of Crenarchaeota in terrestrial habitats studied by 16S RNA surveys and real time PCR. *Environ. Microbiol.* 5 787–797 10.1046/j.1462-2920.2003.00476.x 12919414

[B42] Orem W. H. Voytek M. A. Jones E. J. Lerch H. E. Beates A. L. Corum M. D. (2010). Organic intermediates in the anaerobic biodegrdation of coal to methane under laboratory conditions. *Org. Geochem.* 41 997–1000 10.1016/j.orggeochem.2010.03.005

[B43] Parales R. E. (2010). “Hydrocarbon degradation by betaproteobacteria,” in *Handbook of Hydrocarbon and Lipid Microbiology* ed. Timmis K. (Berlin: Springer) 1715–1724 10.1007/978-3-540-77587-4_121

[B44] Patel G. B. Sprott G. D. (1990). Methanosaeta concilii gen. nov., sp. nov. (“methanothrix concilii”) and methanosaeta thermoacetophila nom. rev., comb. nov. *Int. J. Syst. Bacteriol.* 40 79–82 10.1099/00207713-40-1-79

[B45] Penner T. J. Foght J. M. Budwill K. (2010). Microbial diversity of western canadian subsurface coal beds and methanogenic coal enrichment cultures. *Int. J. Coal Geol.* 82 81–93 10.1016/j.coal.2010.02.002

[B46] Pilloni G. Granitsiotis M. S. Engel M. Lueders T. (2012). Testing the Limits of 454 Pyrotag Sequencing: reproducibility, Quantitative Assessment and Comparison to T-RFLP Fingerprinting of Aquifer Microbes. *PLoS ONE* 7:e40467 10.1371/journal.pone.0040467 PMC3395703 22808168

[B47] Pilloni G. Von Netzer F. Engel M. Lueders T. (2011). Electron acceptor-dependent identification of key anaerobic toluene degraders at a tar-oil-contaminated aquifer by pyro-sip. *FEMS Microbiol. Ecol.* 78 165–175 10.1111/j.1574-6941.2011.01083.x 21385190

[B48] Pruesse E. Peplies J. Glöckner F. O. (2012). Sina: accurate high-throughput multiple sequence alignment of ribosomal rna genes. *Bioinformatics* 28 1823–1829 10.1093/bioinformatics/bts252 22556368 PMC3389763

[B49] Reisfeld A. Rosenberg E. Gutnick D. (1972). Microbial degradation of crude oil: factors affecting the dispersion in sea water by mixed and pure cultures. *Appl. Microbiol.* 24 363–368. 4562475 10.1128/am.24.3.363-368.1972 PMC376525

[B50] Ren H.-Y. Zhang X.-J. Song Z.-Y. Rupert W. Gao G.-J. Guo S.-X. (2011). Comparison of microbial community compositions of injection and production well samples in a long-term water-flooded petroleum reservoir. *PLoS ONE* 6:e23258 10.1371/journal.pone.0023258 PMC3156122 21858049

[B51] Rice D. D. (1993). “Composition and origins of coalbed gas,” in *Studies in Geology,* Vol. 38 *Hydrocarbons from Coal* eds Law B. E. Rice D. D. (Tulsa, OK: American Association of Petroleum Geologists) 159–184.

[B52] Rockne K. J. Chee-Sanford J. C. Sanford R. A. Hedlund B. P. Staley J. T. Strand S. E. (2000). Anaerobic naphthalene degradation by microbial pure cultures under nitrate-reducing conditions. *Appl. Environ. Microbiol.* 66 1595–1601 10.1128/AEM.66.4.1595-1601.2000 10742247 PMC92028

[B53] Rotaru A.-E. Shrestha P. M. Liu F. Shrestha M. Shrestha D. Embree M. (2014). A new model for electron flow during anaerobic digestion: direct interspecies electron transfer to *Methanosaeta* for the reduction of carbon dioxide to methane. *Energ. Environ. Sci.* 7 408–415 10.1039/C3EE42189A

[B54] Schippers A. Neretin L. N. (2006). Quantification of microbial communities in near-surface and deeply buried marine sediments on the peru continental margin using real-time pcr. *Environ. Microbiol.* 8 1251–1260 10.1111/j.1462-2920.2006.01019.x 16817933

[B55] Schloss P. D. Westcott S. L. Ryabin T. Hall J. R. Hartmann M. Hollister E. B. (2009). Introducing mothur: open-source, platform-independent, community-supported software for describing and comparing microbial communities. *Appl. Environ. Microbiol.* 75 7537–7541 10.1128/AEM.01541-09 19801464 PMC2786419

[B56] Schoell M. (1980). The hydrogen and carbon isotopic composition of methane from natural gases of various origins. *Geochim. Cosmochim. Acta* 44 649–661 10.1016/0016-7037(80)90155-6

[B57] Schoell M. (1983). Genetic characterization of natural gases. *AAPG Bull.* 67 2225–2238.

[B58] Shimizu S. Akiyama M. Naganuma T. Fujioka M. Nako M. Ishijima Y. (2007). Molecular characterization of microbial communities in deep coal seam groundwater of northern japan. *Geobiology* 5 423–433 10.1111/j.1472-4669.2007.00123.x

[B59] Shimizu S. Upadhye R. Ishijima Y. Naganuma T. (2011). Methanosarcina horonobensis sp. nov., a methanogenic archaeon isolated from a deep subsurface miocene formation. *Int. J. Syst. Evol. Microbiol.* 61 2503–2507 10.1099/ijs.0.028548-0 21112985

[B60] Siegert M. Cichocka D. Herrmann S. Gründger F. Feisthauer S. Richnow H. H. (2011). Accelerated methanogenesis from aliphatic and aromatic hydrocarbons under iron- and sulfate-reducing conditions. *FEMS Microbiol. Lett.* 315 6–16 10.1111/j.1574-6968.2010.02165.x 21133990

[B61] Sousa D. Z. Alves J. I. Alves M. M. Smidt H. Stams A. J. (2009). Effect of sulfate on methanogenic communities that degrade unsaturated and saturated long-chain fatty acids (lcfa). *Environ. Microbiol.* 11 68–80 10.1111/j.1462-2920.2008.01740.x 18783383

[B62] Steinberg L. M. Regan J. M. (2008). Phylogenetic comparison of the methanogenic communities from an acidic, oligotrophic fen and an anaerobic digester treating municipal wastewater sludge. *Appl. Environ. Microbiol.* 74 6663–6671 10.1128/AEM.00553-08 18776026 PMC2576706

[B63] Steinberg L. M. Regan J. M. (2009). Mcra-targeted real-time quantitative pcr method to examine methanogen communities. *Appl. Environ. Microbiol.* 75 4435–4442 10.1128/AEM.02858-08 19447957 PMC2704849

[B64] Strąpoć D. Mastalerz M. Dawson K. Macalady J. Callaghan A. V. Wawrik F. B. (2011). Biogeochemistry of microbial coal-bed methane. *Annu. Rev. Earth Planet Sci.* 39 617–656 10.1146/annurev-earth-040610-133343

[B65] Strąpoć D. Picardal F. W. Turich C. Schaperdoth I. Macalady J. L. Lipp J. S. (2008). Methane-producing microbial community in a coal bed of the illinois basin. *Appl. Environ. Microbiol.* 74 2424–2432 10.1128/AEM.02341-07 18310416 PMC2293134

[B66] Takai K. Horikoshi K. (2000). Rapid detection and quantification of members of the archaeal community by quantitative pcr using fluorogenic probes. *Appl. Environ. Microbiol.* 66 5066–5072 10.1128/AEM.66.11.5066-5072.2000 11055964 PMC92420

[B67] Tang Y.-Q. Li Y. Zhao J.-Y. Chi C.-Q. Huang L.-X. Dong H.-P. (2012). Microbial communities in long-term, water-flooded petroleum reservoirs with different in situ temperatures in the huabei oilfield, china. *PLoS ONE* 7:e33535 10.1371/journal.pone.0033535 PMC3303836 22432032

[B68] Ulrich G. A. Martino D. Burger K. Routh J. Grossman E. L. Ammerman J. W. (1998). Sulfur cycling in the terrestrial subsurface: commensal interactions, spatial scales, and microbial heterogeneity. *Microb. Ecol.* 36 141–151 10.1007/s002489900101 9688776

[B69] Vieth A. Mangelsdorf K. Sykes R. Horsfield B. (2008). Water extraction of coals – potential for estimating low molecular weight organic acids as carbon feedstock for the deep terrestrial biosphere. *Org. Geochem.* 39 985–991 10.1016/j.orggeochem.2008.02.012

[B70] Voordouw G. Armstrong S. M. Reimer M. F. Fouts B. Telang A. J. Shen Y. (1996). Characterization of 16s rrna genes from oil field microbial communities indicates the presence of a variety of sulfate-reducing, fermentative, and sulfide-oxidizing bacteria. *Appl. Environ. Microbiol.* 62 1623–1629. 8633860 10.1128/aem.62.5.1623-1629.1996 PMC167936

[B71] Wang Q. Garrity G. M. Tiedje J. M. Cole J. R. (2007). Naive bayesian classifier for rapid assignment of rrna sequences into the new bacterial taxonomy. *Appl. Environ. Microbiol.* 73 5261–5267 10.1128/AEM.00062-07 17586664 PMC1950982

[B72] Watanabe K. Kodama Y. Hamamura N. Kaku N. (2002). Diversity, abundance, and activity of archaeal populations in oil-contaminated groundwater accumulated at the bottom of an underground crude oil storage cavity. *Appl. Environ. Microbiol.* 68 3899–3907 10.1128/AEM.68.8.3899-3907.2002 12147488 PMC124022

[B73] Weelink S. A. Van Doesburg W. Saia F. T. Rijpstra W. I. Roling W. F. Smidt H. (2009). A strictly anaerobic betaproteobacterium georgfuchsia toluolica gen. nov., sp. nov. Degrades aromatic compounds with Fe(III), Mn(IV) or nitrate as an electron acceptor. *FEMS Microbiol. Ecol.* 70 575–585 10.1111/j.1574-6941.2009.00778.x 19799633

[B74] Weinbauer M. G. Beckmann C. Höfle M. G. (1998). Utility of green fluorescent nucleic acid dyes and aluminum oxide membrane filters for rapid epifluorescence enumeration of soil and sediment bacteria. *Appl. Environ. Microbiol.* 64 5000–5003. 9835595 10.1128/aem.64.12.5000-5003.1998 PMC90955

[B75] Whiticar M. J. (1999). Carbon and hydrogen isotope systematics of bacterial formation and oxidation of methane. *Chem. Geol.* 161 291–314 10.1016/S0009-2541(99)00092-3

[B76] Whiticar M. J. Faber E. Schoell M. (1986). Biogenic methane formation in marine and freshwater environments: Co2 reduction vs. Acetate fermentation-isotope evidence. *Geochim. Cosmochim. Acta* 50 693–709 10.1016/0016-7037(86)90346-7

[B77] Widdel F. Bak F. (1992). *Gram-Negative Mesophilic Sulfate-Reducing Bacteria. The Prokaryotes: A Handbook on the Biology of Bacteria: Ecophysiology, Isolation, Identification, Application*. New York: Springer-Verlag 3353–3378.

[B78] Wright E. S. Yilmaz L. S. Noguera D. R. (2012). Decipher, a search-based approach to chimera identification for 16s rrna sequences. *Appl. Environ. Microbiol.* 78 717–725 10.1128/AEM.06516-11 22101057 PMC3264099

[B79] Yashiro Y. Sakai S. Ehara M. Miyazaki M. Yamaguchi T. Imachi H. (2011). Methanoregula formicica sp. nov., a methane-producing archaeon isolated from methanogenic sludge. *Int. J. Syst. Evol. Microbiol.* 61 53–59 10.1099/ijs.0.014811-0 19667393

[B80] Yu Y. Breitbart M. Mcnairnie P. Rohwer F. (2006). FastgroupII: a web-based bioinformatics platform for analyses of large 16s rdna libraries. *BMC Bioinformatics* 7:57 10.1186/1471-2105-7-57 PMC1386709 16464253

[B81] Zengler K. Richnow H. H. Rosselló-Mora R. Michaelis W. Widdel F. (1999). Methane formation from long-chain alkanes by anaerobic microorganisms. *Nature* 401 266–269 10.1038/45777 10499582

